# Genetic Variants in *PNPLA3* and Risk of Non-Alcoholic Fatty Liver Disease in a Han Chinese Population

**DOI:** 10.1371/journal.pone.0050256

**Published:** 2012-11-30

**Authors:** Xian-E Peng, Yun-Li Wu, Shao-Wei Lin, Qing-Qing Lu, Zhi-Jian Hu, Xu Lin

**Affiliations:** 1 Key Laboratory of Ministry of Education for Gastrointestinal Cancer, Research Center of Molecular Medicine, Fujian Medical University, Fuzhou, China; 2 Department of Epidemiology and Health Statistics, School of Public Health, Fujian Medical University, Fuzhou, China; 3 Key Laboratory of Tumor Microbiology, Department of Medical Microbiology, Fujian Medical University, Fuzhou, China; 4 Department of Gastroenterology, Union Hospital of Fujian Medical University, Fuzhou, China; Harvard Medical School, United States of America

## Abstract

We investigated the possible association between genetic variants in the Patatin like phospholipase-3 (*PNPLA3*) gene and nonalcoholic fatty liver disease (NAFLD) in a Han Chinese population. We evaluated twelve tagging single-nucleotide polymorphisms (tSNPs) of the *PNPLA3* gene in a frequency matched case–control study from Fuzhou city of China (553 cases, 553 controls). In the multivariate logistic regression analysis, the rs738409 GG or GC, and rs139051 TT genotypes were found to be associated with increased risk of NAFLD, and a significant trend of increased risk with increasing numbers of risk genotype was observed in the cumulative effect analysis of these single nucleotide polymorphisms. Furthermore, haplotype association analysis showed that, compared with the most common haplotype, the CAAGAA**TG**CGTG and CGAAGG**TG**TCCG haplotypes conferred a statistically significant increased risk for NAFLD, while the CGGGAA**CC**CGCG haplotype decreased the risk of NAFLD. Moreover, rs738409 C>G appeared to have a multiplicative joint effect with tea drinking (P<0.005) and an additive joint effect with obesity (Interaction contrast ratio (ICR) = 2.31, 95% CI: 0.7–8.86), hypertriglyceridemia (ICR = 3.07, 95% CI: 0.98–5.09) or hypertension (ICR = 1.74, 95% CI: 0.52–3.12). Our data suggests that *PNPLA3* genetic polymorphisms might influence the susceptibility to NAFLD development independently or jointly in Han Chinese.

## Introduction

Nonalcoholic fatty liver disease (NAFLD) is an emerging epidemic disease with increasing prevalence worldwide [1]. NAFLD refers to a wide spectrum of liver diseases, ranging from fatty liver alone to nonalcoholic steatohepatitis with evidence of liver cell injury, a mixed inflammatory lobular infiltrate, and variable fibrosis [2], [3]. The pathogenesis of NAFLD is multifactorial; it is well-recognized that obesity, insulin resistance, hyperlipidemia, diabetes, and environmental factors influence the development and progression of NAFLD [4], [5]. In addition, genetic factors have long been suspected to play a role in NAFLD development [6], [7], and several genes, including *PPARα*, *PEMT*, *STAT3* and *ABCC2*, have been suggested as potential candidates for either NAFLD susceptibility or disease progression [8], [9], [10], [11].

Patatin like phospholipase-3 (PNPLA3), also known as adiponutrin, belongs to the patatin-like phospholipase family of proteins that contain adipose triglyceride lipase (PNPLA2), the key protein involved in the hydrolysis of triglycerides to diglycerides in adipocytes. PNPLA3 is a transmembrane protein which, in humans, is prominently expressed in hepatocytes and is highly responsive to changes in energy balance [12]. PNPLA3 exhibits hydrolase activity against triglycerides, and variations in PNPLA3 alter its catalytic activity and can lead to triglyceride accumulation in hepatocytes [13], [14]. Recently, genome-wide association studies have suggested that a nonsynonymous sequence variation (rs738409 C>G) that results in an isoleucine to methionine substitution at residue 148 (I148M) in PNPLA3, contributes to differences in hepatic lipid content and the susceptibility to NAFLD [7], [15], [16]. This single nucleotide polymorphism (SNP) was also found to be associated with elevated levels of liver enzymes in healthy subjects, and with disease severity, especially steatosis and fibrosis, in individuals with NAFLD [17], [18], [19], [20]. However, the contribution of PNPLA3 rs738409 genotypes to NAFLD susceptibility in the Han Chinese population is controversial, one study with a group of 112 NAFLD patients showed that the PNPLA3 rs738409 polymorphism was not associated with steatosis [21], while another study with a group of 203 NAFLD patients found that the PNPLA3 rs738409 polymorphism was associated with steatosis [22].

In order to address this issue, we investigated the association of polymorphisms in *PNPLA3* and their predicted haplotypes with the risk for NAFLD in a Han Chinese population. Furthermore, by taking into consideration the fact that NAFLD is a multifactorial disorder, we also studied the possible interactions with environmental and ‘internal’ exposures, such as tea drinking, body mass and other components of the metabolic syndrome.

## Materials and Methods

### Ethics Statement

The use of human blood sample and the protocol in this study were strictly conformed to the principles expressed in the Declaration of Helsinki and were approved by the institutional ethical committees of the Union Hospital of Fujian Medical University and Fujian Medical University. Written informed consent was obtained from all participants before their participation in the study.

### Study Population

We performed a 1∶1 frequency-matched case-control study on NAFLD in a health examination center of Union Hospital of Fujian Medical University, from June of 2008 to May of 2011. The study design has been described in detail elsewhere [23]. Briefly, the study group consisted of 553 unrelated individual NAFLD patients (399 males and 154 females; mean age: 45.33 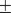 12.48 years) with features of NAFLD and ultrasonographic (US) examinations [24] . Patients with secondary causes of steatosis, including alcohol abuse, total parenteral nutrition, hepatitis B and hepatitis C virus infection, and the use of drugs known to precipitate hepatic steatosis, were excluded. In addition, patients with any of the following diseases were excluded from participation in this study: autoimmune hepatitis, drug-induced liver disease, primary biliary cirrhosis, and primary sclerosing cholangitis. Additionally, 553 healthy individuals (399 males and 154 females; mean age: 43.87±13.00 years) without steatosis by ultrasonography were chosen randomly from local residents during the same study period, who underwent a routine health check and were free of any known major diseases. They were frequency-matched to the NAFLD patients according to sex, age (within 5 years), ethnicity, occupation and area of residence. The ultrasonographic examination of the upper abdominal organs was performed by two experienced physicians using a scanner equipped with a 3.5-MHz transducer (Siemens Adama, Erlangen, Germany). The physicians who performed the ultrasonography were unaware of this study. All enrolled subjects were of Chinese Han ethnicity.

### Demographic and Clinical Data

All subjects underwent a complete physical examination in the morning after an overnight fast. Health examination included anthropometric measurements, abdominal ultrasonography, a questionnaire on health-related behaviors (smoking, and tea drinking), and biochemical determinations. Smokers were defined as subjects who had smoked at least 100 cigarettes during their lifetime and were classified as smokers versus non-smokers. Tea drinkers were defined as individuals who drank at least one cup of green tea per day for more than half a year and classified as drinkers versus non-drinkers. Body measurements including weight and height were measured in a standardized fashion by a trained examiner. Body mass index (BMI) was calculated as follows: weight (kg) divided by height squared (m^2^). Overweightness and/or obesity were defined as having a BMI 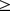
25 kg/m^2^. Three resting blood pressure readings were obtained at 1-min intervals; the second and third systolic and diastolic pressure readings were averaged and used for analysis. Hypertension was defined as systolic blood pressure
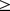
140 mmHg and/or diastolic blood pressure ≥90 mmHg, or if the subject was being prescribed antihypertensive medications. Venous blood samples were drawn just after the anthropometric examinations. Total cholesterol (TC), low-density lipoprotein cholesterol (LDL-C), high-density lipoprotein cholesterol (HDL-C), triglycerides (TG), plasma glucose, and liver function tests were measured by standard clinical laboratory techniques. Reduced HDL-C, hypertriglyceridemia, and raised fasting plasma glucose (FPG) were diagnosed according to the International Diabetes Federation consensus worldwide definition of the metabolic syndrome [25].

### SNP Selection and Genotyping

Genomic DNA was extracted from peripheral blood by standard methods. We selected the tag SNPs by using genotype data obtained from unrelated Han Chinese in Beijing individuals from the HapMap project (http://www.hapmap.ncbi.nlm.nih.gov). We used the pairwise tagging method of the Haploview V4.2 software (Broad institute, Cambridge, MA) to capture SNPs with a minimum minor allele frequency (MAF) of >0.10 and a minimum r^2^ of >0.8 and 12 tag SNPs were selected from the HapMap Project (rs139047, rs738409, rs139051, rs1883350, rs2076208, rs2076212, rs2294918, rs3810622, rs738407, rs9625961, rs734561, rs2006943).

Genotyping was performed with Sequenom Mass Array iPLEX platform (Sequenom Inc., San Diego, CA) using a allele-specific MALDI-TOF mass spectrometry assay. Primers for amplification and extension reactions were designed using Mass Array Assay Design Version 3.1 software (Sequenom Inc.), and SNP genotypes were obtained according to the iPLEX protocol provided by the manufacturer. Laboratory personnel who assessed all genotyping results were blinded to the samples case–control status. Genotyping quality was examined by a detailed quality control procedure consisting of >95% successful call rate, duplicate calling of genotypes, internal positive control samples and Hardy–Weinberg Equilibrium (HWE) testing. The success rate of genotyping was greater than 95%, and the concordance rate was greater than 99% based on 10% of duplicate samples for each SNP.

### Statistical Analysis

Unless otherwise indicated, phenotypic quantitative data were expressed as mean ± standard deviation. To evaluate differences in clinical characteristics between cases and controls, Student’s t-test, Pearson Chi-square or the nonparametric Mann–Whitney U tests were used as appropriate. Hardy-Weinberg equilibrium (HWE) was checked in controls by using the chi-squared test. Unconditional univariate and multivariate logistic regression analyses were performed to obtain the crude and adjusted odds ratios (*ORs*) for risk of NAFLD and their 95% confidence intervals (*CIs*). Age, sex, marriage, education or income, smoking, tea drinking, and clinical characteristics were considered as potential confounders and were also included in the multivariate logistic regression models. Haploview 4.2 software was used to construct the haplotypes [26]. Haplo.stats software package developed using the R language was used to estimate adjusted *ORs* and 95% *CIs* for each haplotype [27]. To assess statistical significance, we performed permutation procedures to correct the *P*-value of single-locus association results for multiple testing. Simulations were run 1,000 times for empirical P-values.

Stratified analysis was used to explore potential gene–environment interactions. We dichotomized the genetic polymorphism by grouping subjects into carriers and non-carriers of the risk genotype. Similarly, ‘internal’ and environmental exposures were dichotomized by appropriate grouping. P-values for multiplicativity were derived from a cross-product term of gene and environment exposure introduced into a multiplicative model. The interaction contrast ratio (ICR) was used to evaluate the potential additive interaction (ICR = OReg – Ore – Org +1; where OReg is the OR for both the genotype and exposure, ORe is the OR for exposure only and ORg is the OR for genotype only). If ICR >0, we concluded that there was a positive additive interaction. Ninety-five percent CIs of ICR were computed according to the method by Hosmer [28], and an ICR can be considered as statistically significant at alpha level 0.05 if its 95% CIs do not include zero. All statistical analyses were performed using R software (version 2.14.1; http://www.r-project.org), unless indicated otherwise, and a two-sided *P*-value <0.05 was considered statistically significant.

### Power Calculations

Power for association studies depends on the sample size, the level of type I error specified, and the genotype frequencies in cases and controls. Genotype frequencies will depend on population frequency of the allele of interest and its effect on disease liability. Genetic Power Calculator [29] was used to estimate the statistical power for the statistically significant results concerning individual polymorphism data. The results showed a power of >95% for rs738409 under a dominant model, and >90% for rs139051 under a recessive model regarding the association with NAFLD found for the allele frequencies and sample size in the present study. Power calculations for the statistically significant results concerning gene- environment interactions were computed according to the method by Foppa [30] , and the results showed a power of >90% for the statistically significant interactions between rs738409 C>G and external or ‘internal’ environmental exposure.

## Results

### Study Population Characteristics

The detail data of behaviors, clinical features, anthropometric variables and laboratory findings at diagnosis available in patients and healthy controls had been previously described [23]. Briefly, no statistically significant differences (P>0.05) were found between patients with NAFLD and controls, in terms of age, sex, marriage, education, smoking or income status. Compared to controls, NAFLD patients had a lower percentage of tea drinkers, as well as higher occurrence of most of the risk factors of the metabolic syndrome, including elevated blood pressure, BMI, FPG, TC, TG, LDL-C and decreased HDL-C. In addition, levels of alanine transaminase (ALT) and aspartate transaminase (AST) were significantly higher in patients with NAFLD, compared to controls (P<0.05).

### Association between Individual Genotypes and Risk of NAFLD

The SNP information, observed allele frequencies and Hardy–Weinberg test results are presented in Table S1. Twelve *PNPLA3* SNPs were genotyped with a mean success rate of 98.1% (range 95.4 to 100%) and all the genotype distributions did not deviate from Hardy–Weinberg equilibrium. In the single allelic analysis, tests for associations between NAFLD and the tag SNPs showed significant differences only for rs738409, and the association remained significant after multiple comparison correction by permutation tests (P<0.005). The risk allele (G-allele) frequency of rs738409 in the control subjects was 0.34 and 0.42 in the NAFLD patients. After adjustment for age, sex, income, marriage, education, smoking, tea drinking, BMI and other clinical features, the genotype distributions of rs738409 and rs139051 showed significant associations with NAFLD in the multivariate logistic regression model (Table 1). A significant risk effect on NAFLD was found to be associated with the genotypes of rs738409. Specifically, compared to the subjects with the CC homozygous alleles, carriers of heterozygous CG and homozygous GG genotypes had a significantly increased risk of NAFLD (adjusted OR = 1.39, 95% CI: 1.03–1.87 for CG genotype; adjusted OR = 2.25, 95% *CI*: 1.46–3.45 for GG genotype) in an allele dose-response manner (adjusted *P*_Trend_ = 0.0004). This association was further revealed using a dominant model (GG+CG vs. CC, adjusted OR: 1.74; 95% CI: 1.28–2.38, P<0.001). In addition, we also found that homozygous TT genotype for rs139051 was associated with a significantly increased risk of NAFLD (adjusted OR = 1.67, 95% CI = 1.02–2.73), compared with the wild-type CC homozygous genotype; this association was better demonstrated under a recessive model (TT vs. CC+CT, adjusted OR: 1.52; 95% CI: 1.11–2.08, P<0.01). We found no significant associations between others SNPs and NAFLD in the multivariate logistic regression models (data not shown).

**Table 1:** Association between the genotypes of rs738409 and rs139051 and risk of NAFLD.

Polymorphism ID no.	Genotypes	Cases	Controls	Crude OR and 95%CI	Adjusted OR and 95% CI^a^	P-value^a^
		n (%)	n (%)			
rs738409						
	CC	183 (33.15)	235 (42.50)	1.00 (Reference)	1.00 (Reference)	–
	CG	276 (50.00)	259 (46.84)	1.37 (1.06–1.77)	1.39 (1.03–1.87)	0.010
	GG	93 (16.85)	59 (10.67)	2.02 (1.39–2.96)	2.25 (1.46–3.45)	0.001
*P* for trend						0.0004
CG+GG vs. CC				1.49 (1.17–1.90)	1.77 (1.29–2.41)	0.001
rs139051						
	CC	68 (12.64)	71 (13.15)	1.00 (Reference)	1.00 (Reference)	**-**
	TC	257 (47.77)	270 (50.00)	1.02 (0.70–1.48)	0.99 (0.68–1.45)	0.969
	TT	213 (39.59)	199 (36.85)	1.16 (0.79–1.70)	1.67 (1.02–2.73)	0.039
*P* for trend						0.150
TT vs. CC+TC				1.12 (0.88–1.44)	1.52(1.11–2.08)	0.009

n: number of individuals; OR (odds ratio) was determined using logistic regression and adjusted for sex, age, body mass index and other clinical characteristics; CI: confidence interval.

a P-value based on the Wald test.

### Association of Phenotype with Different rs738409 and rs139051 Genotypes

To investigate whether the genotypes of rs738409 or rs139051 were associated with clinical parameters, we compared age, gender, BMI, FPG, TC, TG, HDL-C, LDL-C, AST, and ALT between different genotypes of rs738409 and rs139051 in the NAFLD and control subjects. We did not observe any association of rs738409 variant with age, gender, BMI index, TG, TC, HDL-C, LDL-C, or FPG levels both in NAFLD patients and controls (P>0.05). In contrast, AST (P<0.05) levels were significantly higher in NAFLD patients harboring the risk allele (details in Table S2). Nevertheless, the T-allele of rs139051 was associated with significantly decreased BMI and TG levels (P<0.05) in the NAFLD patients. We did not observe any association of rs139051 variant with age, gender, BMI index, TG, HDL-C and LDL-C, FPG, AST, or ALT levels in controls (details in Table S3).

### Combined Association between rs738409 and rs139051 and Risk of NAFLD

We further checked the possible cumulative risk effect of rs738409 and rs139051 on risk of NAFLD, finding that NAFLD patients tended to have more risk genotypes than controls (Table 2). When using those without variant risk genotype as references, subjects with two risk genotypes had a 2.40-fold (95% CI: 1.58–3.65) excess risk for NAFLD. The risk for NAFLD increased progressively as the number of risk genotypes increased (*P*_trend_ <0.001). When the combined genotypes were dichotomized into two groups (i.e., 0 risk genotypes vs. 1 to 2 risk genotypes), we found that the combined genotype was associated with a significantly increased risk of NAFLD compared with those without risk genotypes (adjusted OR = 1.70, 95% CI :1.20–2.40).

**Table 2:** Cumulative effect of adverse genotypes in PNPLA3 rs738409 and rs139051 on NAFLD.

No of risk genotypes^a^	Cases	Controls	Crude OR and 95%CI	Adjusted OR and 95%CI^b^	*P* c
	n (%)	n (%)			
0	126 (42.86)	168 (57.14)	1.00(Reference)	1.00(Reference)	–
1	248 (51.24)	236 (48.76)	1.40(1.05–1.88)	1.38(0.95–2.01)	0.090
2	164 (54.67)	136 (45.33)	1.61(1.16–2.22)	2.40(1.58–3.65)	<0.001
*P* for trend			1.27(1.08–1.49)	1.55(1.26–1.91)	0.001
1 or 2	412(52.55)	372(47.45)	1.48(1.13–1.94)	1.70(1.20–2.40)	0.003

a Risk genotypes were GC/GG for rs734089 and TT for rs139051.

b Individuals with no risk genotype were set as the reference group. OR (odds ratio) was determined using logistic regression and adjusted for sex, age, body mass index and other clinical characteristics; CI: confidence interval.

c *P*-value based on the Wald test;

### Association between Haplotypes and Risk of NAFLD

LD pattern among *PNPLA3* polymorphisms was investigated to determine haplotype blocks in our study population and two independent haplotype blocks were detected within the investigated region. One region contained rs738407, rs734561, and rs2006943) and the other region contained rs1883350, rs2076208, rs3810622 and rs2294918. The SNPs that were significantly associated with NAFLD (rs139051 and rs738409) were not located on block 1 or block 2.

The results of the association between the PNPLA3 haplotype and the risk of NAFLD were listed in Table 3. There was no haplotype both in block1 and block2 found to be associated with the risk of NAFLD. However, global haplotype association analysis showed that after adjustment for age, sex, education, marriage, smoking, tea drinking, income, BMI and other clinical characteristics, individuals carrying the haplotypes CAAGAATGCGTG (adjusted OR = 1.63, 95% CI: 1.03–2.58) and CGAAGGTGTCCG (adjusted OR = 2.22, 95% CI: 1.05–4.71) conferred a statistically significant increased risk for NAFLD, while individuals carrying the haplotype CGGGAACCCGCG conferred a statistically significant decreased risk for NAFLD (adjusted OR = 0.45, 95% CI: 0.21–0.96), compared with those carrying the most common haplotype CGAAGGTCTCCG. Interestingly, the only difference between the risk haplotype CGAAGGTGTCCG and the most common haplotype CGAAGGTCTCCG was the SNP rs738409 G allele, suggesting that the rs738409 G allele may be the risk allele for NAFLD, which was consistent with the individual SNP association analysis.

**Table 3:** Associations between risk of NAFLD and frequencies of inferred haplotypes on the basis of the observed genotypes in NAFLD cases and controls.

Block	Haplotypes	Total frequency	Haplotype frequencies		P-value^d^	OR and 95% CI^e^
			Case	Control		
Block1 a	AGG	0.437	0.433	0.441	0.697	1.00 (Reference)
	GAA	0.353	0.353	0.354	0.956	1.00(0.82–1.22)
	GGG	0.126	0.129	0.123	0.655	1.07(0.81–1.41)
	GGA	0.083	0.085	0.082	0.796	1.05(0.75–1.46)
Block2 b	CGTG	0.386	0.390	0.383	0.728	1.00 (Reference)
	TCCG	0.323	0.328	0.318	0.595	1.01(0.82–1.25)
	TGTA	0.177	0.185	0.170	0.371	1.06(0.82–1.36)
	CGCG	0.067	0.060	0.074	0.179	0.72(0.50–1.06)
	TGCG	0.024	0.019	0.029	0.118	0.67(0.37–1.23)
	TGTG	0.015	0.010	0.020	0.059	0.52(0.32–1.15)
Total^c^	CGAAGGTCTCCG	0.120	0.118	0.125	0.823	1.00 (Reference)
	CAAAGGCCTGTA	0.088	0.088	0.092	0.796	0.95 (0.62–1.44)
	CAAGAATGCGTG	0.076	0.098	0.059	0.020	1.63 (1.03–2.58)
	CGAGAATGCGTG	0.054	0.042	0.064	0.420	0.69 (0.39–1.22)
	CAAAGGTCTCCG	0.038	0.034	0.046	0.075	0.58 (0.30–1.14)
	CGGGAACCTCCG	0.038	0.037	0.042	0.703	0.94 (0.55–1.60)
	CAAGAATCCGTG	0.035	0.032	0.036	0.782	0.95 (0.52–1.71)
	CGAGGGTGCGTG	0.035	0.039	0.028	0.572	1.17 (0.64–2.15)
	CGAAGGTGTCCG	0.031	0.043	0.018	0.011	2.22 (1.05–4.71)
	CGAGAATCCGTG	0.028	0.028	0.029	0.569	0.99 (0.50–1.97)
	CGAGGATGCGTG	0.027	0.032	0.020	0.141	1.60 (0.84–3.06)
	CAAGGATGCGTG	0.024	0.026	0.024	0.515	1.24 (0.60–2.57)
	CGAAGGCCTGTA	0.023	0.026	0.017	0.669	1.29 (0.55–3.04)
	CGGGAACCCGCG	0.023	0.016	0.033	0.013	0.45 (0.21–0.96)
	CAAAGGCGTGTA	0.022	0.025	0.019	0.294	1.38 (0.65–2.90)
	rare haplotypes f	0.360	0.341	0.367	0.692	1.13(0.72–2.08)

a In the order of rs738407, rs734561, and rs2006943);

b In the order of rs1883350, rs2076208, rs3810622 and rs2294918;

c In the order of rs2076212, rs139047, rs9625961, rs738407, rs734561, rs2006943, rs139051, rs738409, rs1883350, rs2076208, rs3810622, and rs2294918.

d p value was adjusted by multiple comparisons corrections; e:OR (odds ratio) was adjusted for sex, age, body mass index and other clinical characteristics; CI: confidence interval.

f All other haplotypes that had frequency <2% in either cases or controls were pooled.

### Possible Interactions of the PNPLA3 Polymorphism and Environmental or ‘Internal’ Exposures on NAFLD

We also explored combined effects of the *PNPLA3* polymorphisms and some environmental or ‘internal’ exposures. No significant interactions (both multiplicative and additive) between rs139051 T>C and tea drinking, BMI index, reduced HDL-C, hypertriglyceridemia, hypertension, and elevated FPG on NAFLD risk were observed (Table 4). However, rs738409 polymorphism appeared to have a multiplicative joint effect with tea drinking (P<0.005) and an additive joint effect with obesity (ICR = 2.31, 95% CI: 0.7–8.86), hypertriglyceridemia (ICR = 3.07, 95% CI: 0.98–5.09) or hypertension (ICR = 1.74, 95% CI: 0.52–3.12) in identifying NAFLD risk (Table 4).

**Table 4:** Combined effects^a^ of *PNPLA3* polymorphisms and environmental and ‘internal’ exposures on the risk of NAFLD.

Exposures	rs139051 genotype		rs738409 genotype	
	CC/TC	TT	CC	CG/GG
Tea drinking				
Yes	1.00 (Reference)	1.04 (0.73–1.48)	1.00 (Reference)	1.21 (0.80–1.82)
No	2.29 (1.67–3.13)	2.70 (1.89–3.86)	1.42 (0.89–2.25)	3.55 (2.37–5.33)
*P-value* formultiplicative interaction^a^	0.37		0.002	
ICR (95%CI)^a^	0.37 (−0.30–1.04)		1.92 (1.40–2.56)	
BMI				
Normal or lean	1.00 (Reference)	1.58 (1.12–2.21)	1.00 (Reference)	1.85 (1.31–2.61)
Overweight or obese	10.25 (6.64–15.84)	8.95 (5.38–14.88)	8.95 (5.33–15.03)	12.11 (7.58–19.36)
*P-value* for multiplicative interaction^a^	0.22		0.36	
ICR (95%CI)^a^	−1.88 (−6.87–2.17)		2.31 (0.70–8.86)	
Hypertriglyceridaemia				
No	1.00 (Reference)	1.25 (0.93–1.68)	1.00 (Reference)	1.55 (1.14–2.11)
Yes	4.06 (2.86–5.76)	5.82 (3.51–9.65)	3.81 (2.47–5.88)	7.43 (4.86–11.34)
*P-value* formultiplicative interaction a	0.32		0.89	
ICR (95%CI)^a^	1.51 (−0.62–3.50)		3.07 (0.98–5.09)	
Reduced HDL-C				
No	1.00 (Reference)	1.16 (0.89–1.52)	1.00 (Reference)	1.55 (1.18–2.03)
Yes	3.69 (2.33–5.82)	4.43 (2.33–8.42)	3.74 (2.12–6.61)	5.73 (3.43–9.55)
*P-value* for multiplicative interaction^a^	0.51		0.76	
ICR (95%CI)^a^	0.58 (−1.63–2.84)		1.44 (−0.86–3.77)	
Hypertension				
No	1.00 (Reference)	1.21 (0.93–1.57)	1.00 (Reference)	1.35 (1.04–1.76)
Yes	2.97 (1.80–4.90)	1.94 (1.10–3.41)	1.49 (0.80–2.79)	3.58 (2.21–5.80)
*P-value* for multiplicative interaction^a^	0.26		0.17	
ICR (95%CI)^a^	−1.24 (−2.56–0.036)		1.74 (0.52–3.12)	
Raised FPG				
No	1.00 (Reference)	1.12 (0.86–1.45)	1.00 (Reference)	1.58 (1.21–2.05)
Yes	1.95 (1.22–3.12)	2.79 (1.43–5.45)	2.54 (1.41–4.58)	2.91 (1.74–4.88)
*P-value* for multiplicative interaction^a^	0.83		0.36	
ICR (95%CI)^a^	0.72 (−0.72–2.22)		−0. 21 (−1.56–1.32)	

a Included ORs and their corresponding 95% CIs.

b Adjusted for age, sex, education, marriage, smoking, income status, and other confounding characteristics.

## Discussion

In this study, we selected 12 tag SNPs in the *PNPLA3* gene and examined these variants and their haplotypes on NAFLD in a Han Chinese population. Our study results indicated that individuals carrying the rs738409 G-allele may predispose them to NAFLD. Compared with subjects with the CC homozygous genotype, carriers of heterozygous CG and homozygous GG genotypes had a significantly increased risk of NAFLD in an allele dose-response manner. In addition, rs738409 C>G appeared to have a multiplicative joint effect with tea drinking and an additive joint effect with obesity, hypertriglyceridemia or hypertension in identifying NAFLD risk, respectively. We also found that the rs139051 TT genotype was associated with a statistically significantly increased risk of NAFLD. The cumulative effect of the rs139051 and rs738409 SNPs was estimated and a significant trend of increased risk with increasing numbers of risk genotypes was observed. Furthermore, the results of haplotype analysis suggested that the haplotypes CAAGAATGCGTG and CGAAGGTGTCCG increased the risk for NAFLD, while haplotype CGGGAACCCGCG decreased risk for NAFLD, compared with the most common haplotype CGAAGGTCTCCG. Therefore, the current data showed that *PNPLA3* genetic polymorphisms might influence the susceptibility to developing NAFLD independently or jointly in the Han Chinese population.

PNPLA3 is a membrane bound protein expressed primarily in adipose tissue and liver [31], [32]. The expression level of PNPLA3 is nutritionally regulated [32], [33], [34], and increases with obesity [35]. The physiological function of PNPLA3 is not fully understood; though it has been shown to have close homology with adipose triglyceride lipase [36], and seems to possess both triglyceride lipase and acyl coenzyme A–independent transacylase activity [37]. Moreover, it has been recently shown that the PNPLA3 may also have a role in adipogenesis, being up-regulated during the differentiation of white adipocytes [38]. Recently, two genome wide association studies identified a polymorphism in *PNPLA3* gene, rs738409 C>G, encoding for the I148M protein variant, as the strongest genetic determinant of hepatic steatosis and increased ALT levels [15], [16]. Subsequently, other studies confirmed this association [17], [18], [19], [20]; however, inconsistent findings were also reported in different populations, including absence of association with hepatic steatosis in a pediatric population [17], Hispanic Americans [15], and white (non-Hispanic) women residing in the United States [39]. Reasons for the previous inconsistent findings may include small sample sizes, different race/ethnicity, inappropriate study design, different linkage disequilibrium with other SNPs and the different prevalence of NAFLD in a variety of populations. In the present study, we found that compared with the rs738409 CC homozygote, the rs738409 CG heterozygote and GG homozygote were associated with a significantly increased risk of developing NAFLD in a Han Chinese population, which is consistent with the previous report by Li et al. [22]. No associations were observed between *PNPLA3* genotype and plasma concentrations of TG, TC, FPG, HDL-C, LDL-C or BMI index which has been confirmed by other reports, either in the general population or specifically in individuals with NAFLD [19]. These findings suggested that the G-allele of the *PNPLA3* rs738409 SNP increased the susceptibility to NAFLD independent of BMI, plasma lipids, and PFG in the Han Chinese population.

To date, the exact role of the *PNPLA3* I148M polymorphism in NAFLD is unclear, although mechanisms by which variation in PNPLA3 may affect liver triglyceride content have been hypothesized. In vitro rodent studies have shown that the 148M allele encodes for a loss-of-function variant that predisposes to steatosis by decreasing triglyceride hydrolysis in hepatocytes [13]. Recent studies found that subjects carrying the rs738409 PNPLA3 G-allele showed smaller adipocytes [18] and suggested that this allele may contribute to the development of hepatic steatosis by modulating adipocyte size, which reflects the amount of lipid able to be stored in the subcutaneous fat depot. Thus, limited expandability of the subcutaneous adipose tissue depot has been considered as a possible factor leading to fat accumulation in ectopic tissues and organs such as the liver [40]. As such, while these factors may explain the association between the *PNPLA3* I148M polymorphism and NALFD, a definitive mechanism has not yet been established and merits further study.

Whether *PNPLA3* rs738409 genotypes affect ALT and AST levels is not clear. In obese subjects with NAFLD of Caucasian [35], [41], Hispanic [15], or Italian heritage [42], the G-allele of rs738409 is significantly associated with increased AST or ALT levels. However, in the African American, European American [15], and German populations [43], rs738409 is not associated with either raised ALT or AST levels. In our study, the association between the G-allele of rs738409 and increased levels of ALT were observed only in the NAFLD group. Reasons for the inconsistent findings may include population bias, i.e., whether the subjects included adults or children, patients with disease (NAFLD or obesity), and the prevalence and severity of NAFLD in the various study populations.

In the current study, increased risk for NAFLD was also noted for rs139051 C>T variation using a recessive model. The rs139051 is an intronic polymorphism; although its functional consequences is less intuitive, intronic polymorphisms of other genes have been reported to be associated with a variety of chronic diseases including NAFLD [11], essential hypertension [44] and type II diabetes [45]. In addition, immunoprecipitation and gene expression experiments suggest that up to 40% of transcription factor binding sites are located within introns [46], implying the important role of introns in gene regulation. As such, the exact molecular mechanism(s) of the rs139051 C>T SNP involved in increased NAFLD risk requires further investigation. Given the known association between NAFLD and insulin resistance and obesity, we also tested for an association of PNPLA3 rs139051 polymorphisms with FPG, plasma lipids and BMI. We found that similar to *PNPLA3* rs738409 G allele [19], the rs139051 T variant allele is associated with a significantly decreased total TG and TC in NAFLD patients. This results suggested that either rs738409 C>G or PNPLA3 rs139051 C>T confer a susceptible genetic background for NAFLD without affecting the severity of FPG, plasma lipids and obesity.

Accumulating evidence has shown that the effect of single genetic variations may be dependent on other genetic variations (gene–gene interaction) or environmental factors (gene–environment interaction) [47]. In this regard, it is conceivable that NAFLD is the result of interactions between multiple genetic variations and environmental factors. In order to interpret the locus–locus and gene–environment interaction, the cumulative effect of rs738409 and rs139051 polymorphisms and the interaction effects between gene and the environmental or ‘internal’ exposures were also estimated. Our findings revealed that individuals with two risk alleles were at significantly higher risk of NAFLD than those without a risk allele, and a significant trend of increased risk with increasing numbers of risk alleles was observed, suggesting that the accumulation of risk alleles in *PNPLA3* gene may increase the NAFLD risk. Furthermore, our results also suggest that rs738409 polymorphism appears to have a multiplicative joint effect with tea drinking and an additive joint effect with obesity, hypertriglyceridemia or hypertension in identifying NAFLD risk, respectively. Obesity, hypertriglyceridemia and hypertension are established risk factors for NAFLD and such an interaction may be expected. Previous studies suggest that green tea consumption decreases mortality from cardiovascular disease [48] and that it may protect against liver disorders [49]. Consistently, our data showed that tea drinking was a protective factor for NAFLD. Notably, this study found a possible interaction between rs738409 polymorphism and tea drinking. Among individuals carrying rs738409 CG/GG genotypes, the risk of NAFLD in non-tea drinkers was almost 3-fold that of tea drinkers. This result indicates that tea consumption might have a potentially beneficial role in NAFLD prevention among individuals with rs738409 CG/GG genotypes. Intervention trials are needed in the future to provide more convincing evidence. The mechanisms by which tea drinking and rs738409 C>G polymorphism interact to affect hepatic steatosis are not known. Previous studies have showed that tea polyphenols were able to decrease the expression of hepatic lipogenic genes, as well as anti-inflammatory and anti-oxidative genes which are also involved in lipid metabolism. Moreover, PNPLA3 also appears to be part of a family of enzymes that affect lipid metabolism. These results warrant further studies to unravel the mechanisms explaining the relationship among tea drinking, *PNPLA3* genotype, and hepatic steatosis.

The strengths of our study included the adoption of a haplotype-based analysis to assess the joint effects of the *PNPLA3* genetic variations on NAFLD. As NAFLD is seemingly a disease involving multiple SNPs in multiple genes, a haplotype-based analysis may be more powerful than analyzing a single locus. In our present study, although all the SNPs except for rs738409 were not associated with NAFLD risk in the single-locus analysis, haplotype analysis showed that two haplotypes were significantly associated with the risk of NAFLD, suggesting that these variants may play a joint role in the development of NAFLD. Also, because of the relatively large sample size, we might have adequate statistical power to detect a modest excess risk. Based on our sample size and the minor allele frequency, the power of detecting an OR of 1.5 at a two-sided α = 0.05 was >90%. Finally, the strong interaction between tea drinking and rs738409 C>G polymorphism could potentially provide important clues that can be used to devise prevention programs and strategies for limiting NAFLD development in individuals carrying the rs738409 G variant.

Several limitations in our study need to be addressed. First, recall bias was inevitable in this case–control study. However, it would not affect the genotype data. Thus recall bias is of less concern in studying genetic-disease associations. Second, the diagnosis of NAFLD was primarily based on ultrasonographic findings; however, this limitation is unavoidable, because for ethical reasons diagnosis of NAFLD based on a more invasive technique such as liver biopsy would be unacceptable. Finally, in the present study, the tag SNPs were chosen to maximize SNP tagging for genetic variation rather than for the functionality of the *PNPLA3* gene, and further mechanistic studies are needed to determine how these SNPs, especially intronic rs139051 C >T, influence protein function.

In conclusion, our study shows that some representative genetic variants in PNPLA3 may modulate the risk of NAFLD in the Han Chinese population. Furthermore, these variants in combination with some environmental factors and ‘internal’ exposures could play a joint role in the development of NAFLD. Studies with ethnically diverse populations and functional evaluation are warranted to confirm our findings.

## Supporting Information

Table S1 Description of SNPs identified for *PNPLA3*.
(DOC)

Table S2 Comparison of various quantitative phenotypes among the different genotypes at rs738409 in *PNPLA3* in patients with NAFLD and control subjects.
(DOC)

Table S3 Comparison of various quantitative phenotypes among the different genotypes at rs139051 in *PNPLA3* in patients with NAFLD and control subjects.
(DOC)
