# Supplementary material for: Genetic Variants in PNPLA3 and Risk of Non-Alcoholic Fatty Liver Disease in a Han Chinese Population
Source: PLoS One. 2012 Nov 30;7(11):e50256. doi: 10.1371/journal.pone.0050256 (PMC3511464; doi:10.1371/journal.pone.0050256)
Supplement: Table S1 — Description of SNPs identified for PNPLA3. (DOC) [file pone.0050256.s001.doc]

**Table S1. Description of SNPs identified for *PNPLA3*.**

| NCBI SNP reference*^a^* | Chromosome position*^b^* | Genic location | Allele*^c^* | MAF | | | *P* value for |
| --- | --- | --- | --- | --- | --- | --- | --- |
|  |  |  |  | Controls | Cases | *P^d^* | HWF |
| rs738409 | 44324727 | exon 3 | C/G | 0.341 | 0.413 | 0.005 | 0.380 |
| rs139047 | 44323074 | intron 2 | G/A | 0.447 | 0.451 | 0.849 | 1.000 |
| rs139051 | 44324676 | intron 2 | T/C | 0.365 | 0.381 | 0.435 | 0.203 |
| rs1883350 | 44328043 | intron 3 | T/C | 0.451 | 0.462 | 0.670 | 0.172 |
| rs2076208 | 44331060 | intron 5 | G/C | 0.34 | 0.324 | 0.441 | 0.171 |
| rs2076212 | 44322970 | exon 2 | C/A | 0.047 | 0.050 | 0.676 | 0.794 |
| rs2294918 | 44342116 | exon 9 | G/A | 0.197 | 0.178 | 0.270 | 0.249 |
| rs3810622 | 44338134 | intron 7 | T/C | 0.414 | 0.426 | 0.548 | 0.142 |
| rs738407 | 44323955 | intron 2 | G /A | 0.434 | 0.439 | 0.727 | 0.301 |
| rs9625961 | 44323219 | intron 2 | G/ A | 0.16 | 0.179 | 0.238 | 0.946 |
| rs734561 | 44324104 | intron 2 | G/A | 0.353 | 0.353 | 0.949 | 0.600 |
| rs2006943 | 44324181 | intron 2 | G/A | 0.432 | 0.432 | 0.984 | 0.183 |

HWE: Hardy-Weinberg equilibrium; MAF: minor allele frequency;

*a:* single nucleotide polymorphisms on NCBI Reference Assembly; *b*: SNP position in the NCBI dbSNP database (<http://www.ncbi.nlm.nih.gov/SNP>); *c*: major/minor allele;

*d*: After correcting for multiple testing by Haploview program using 1,000 permutations.
