# Supplementary material for: Genetic Variants in PNPLA3 and Risk of Non-Alcoholic Fatty Liver Disease in a Han Chinese Population
Source: PLoS One. 2012 Nov 30;7(11):e50256. doi: 10.1371/journal.pone.0050256 (PMC3511464; doi:10.1371/journal.pone.0050256)
Supplement: Table S2 — Comparison of various quantitative phenotypes among the different genotypes at rs738409 in PNPLA3 in patients with NAFLD and control subjects. (DOC) [file pone.0050256.s002.doc]

**Table S2. Comparison of various quantitative phenotypes among the different genotypes at rs738409 in *PNPLA3* in patients with NAFLD and control subjects.**

| Quantitative |  | NAFLD |  |  |  |  | Control |  |  |
| --- | --- | --- | --- | --- | --- | --- | --- | --- | --- |
| phenotype | CC (n=183) | CG (n=276) | GG (n=93) | *P^a^* |  | CC (n=235 | CG (n=259) | GG (n=59) | *P^a^* |
| Age (year) | 44.94±12.45 | 45.82±12.29 | 45.25±13.95 | 0.75 |  | 44.73±12.49 | 43.09±13.47 | 44.10±13.02 | 0.37 |
| BMI (kg/m^2^) | 25.72±3.29 | 25.27±2.51 | 25.32±3.04 | 0.25 |  | 22.31±2.33 | 22.12±2.23 | 22.44±2.38 | 0.52 |
| FBS (mg/dL) | 103.34±21.43 | 103.43±23.14 | 101.98±16.66 | 0.85 |  | 99.65±23.34 | 98.34±22.01 | 94.82±10.23 | 0.31 |
| TC(mg/dL) | 204.97±39.52 | 209.10±41.17 | 215.39±37.02 | 0.12 |  | 200.09±41.55 | 194.84±43.31 | 188.89±32.44 | 0.13 |
| TG(mg/dL) | 171.22±110.84 | 162.82±91.13 | 165.62±141.74 | 0.72 |  | 114.70±64.19 | 106.22±54.41 | 99.47±44.03 | 0.11 |
| HDL-C (mg/dL) | 52.36±30.05 | 52.43±14.66 | 54.00±14.45 | 0.80 |  | 62.97±35.88 | 61.03±15.44 | 61.80±16.08 | 0.71 |
| LDL-C (mg/dL) | 133.84±47.04 | 135.44±41.95 | 139.91±39.34 | 0.54 |  | 124.56±41.61 | 119.01±38.01 | 112.31±35.67 | 0.07 |
| SBP (mm Hg) | 127.07±13.28 | 129.12±13.27 | 128.39±9.74 | 0.24 |  | 119.70±12.73 | 120.42±10.91 | 120.64±12.10 | 0.75 |
| DBP (mm Hg) | 78.89±10.70 | 80.37±10.38 | 80.99±9.08 | 0.19 |  | 73.05±10.59 | 73.63±9.43 | 74.03±8.97 | 0.72 |
| AST (IU/L) | 24.46±12.15 | 24.16±10.36 | 30.48±40.58 | 0.02 |  | 25.37±47.28 | 22.88±11.93 | 24.17±18.57 | 0.84 |
| ALT (IU/L) | 35.31±32.66 | 34.34±43.85 | 36.94±30.65 | 0.85 |  | 30.33±104.41 | 28.08±44.55 | 24.08±28.10 | 0.70 |

Data are represented as the mean ± SD. *a*: *P*-values were analyzed using the Kruskal–Wallis test in each group of NAFLD and control subjects.
