# Supplementary material for: Genetic Variants in PNPLA3 and Risk of Non-Alcoholic Fatty Liver Disease in a Han Chinese Population
Source: PLoS One. 2012 Nov 30;7(11):e50256. doi: 10.1371/journal.pone.0050256 (PMC3511464; doi:10.1371/journal.pone.0050256)
Supplement: Table S3 — Comparison of various quantitative phenotypes among the different genotypes at rs139051 in PNPLA3 in patients with NAFLD and control subjects. (DOC) [file pone.0050256.s003.doc]

**Table S3. Comparison of various quantitative phenotypes among the different genotypes at rs139051 in *PNPLA3* in patients with NAFLD and control subjects.**

| Quantitative |  | NAFLD |  |  |  |  | Control |  |  |
| --- | --- | --- | --- | --- | --- | --- | --- | --- | --- |
| phenotype | TT (n=213) | TC (n=257) | CC (n=68) | *P^a^* |  | TT (n=197 | TC (n=270) | CC (n=73) | *P^a^* |
| Age (year) | 45.72±13.31 | 45.55±12.45 | 44.82±11.55 | 0.88 |  | 43.45±13.63 | 43.78±12.80 | 45.22±12.05 | 0.61 |
| BMI (kg/m^2^) | 24.97±2.68 | 25.77±2.69 | 25.46±2.46 | 0.01 |  | 22.26±2.41 | 22.16±2.19 | 22.17±2.27 | 0.88 |
| FBS (mg/dL) | 101.62±18.48 | 104.48±25.03 | 103.72±18.10 | 0.36 |  | 96.98±16.61 | 99.25±23.11 | 97.11±8.77 | 0.41 |
| TC (mg/dL) | 204.88±38.19 | 212.12±41.31 | 206.03±41.99 | 0.13 |  | 193.70±40.68 | 197.43±38.52 | 194.22±35.73 | 0.56 |
| TG(mg/dL) | 156.32±101.23 | 164.62±101.73 | 199.31±141.93 | 0.02 |  | 105.28±58.39 | 110.57±56.27 | 115.01±65.40 | 0.42 |
| HDL-C (mg/dL) | 53.58±28.34 | 52.79±14.96 | 48.83±13.24 | 0.27 |  | 62.24±15.81 | 61.02±15.47 | 65.90±60.09 | 0.37 |
| LDL-C(mg/dL) | 134.45±47.04 | 137.48±40.78 | 130.23±42.05 | 0.44 |  | 116.84±41.82 | 122.13±38.26 | 122.39±37.69 | 0.32 |
| SBP (mm Hg) | 127.85±12.10 | 129.07±13.54 | 127.07±12.53 | 0.41 |  | 120.63±11.58 | 119.99±11.89 | 118.19±11.93 | 0.32 |
| DBP (mm Hg) | 79.86±9.77 | 80.36±10.86 | 78.82±10.02 | 0.54 |  | 73.47±9.50 | 73.83±10.20 | 71.05±9.49 | 0.10 |
| AST (IU/L) | 24.89±11.07 | 26.31±26.22 | 23.73±10.12 | 0.92 |  | 25.12±28.86 | 32.30±104.07 | 23.50±16.66 | 0.50 |
| ALT (IU/L) | 35.14±25.50 | 35.97±49.84 | 33.96±22.16 | 0.56 |  | 23.77±15.26 | 24.99±44.43 | 21.46±6.25 | 0.71 |

Data are represented as the mean ± SD. *a*: *P*-values were analyzed using the Kruskal–Wallis test in each group of NAFLD and control subjects.
